# Adding L-carnitine to antagonist ovarian stimulation doesn’t improve the outcomes of IVF/ ICSI cycle in patients with polycystic ovarian syndrome: a double-blind randomized clinical trial

**DOI:** 10.1186/s13048-023-01319-7

**Published:** 2024-01-09

**Authors:** Maryam Hafezi, Arezoo Arabipoor, Firouzeh Ghaffari, Samira Vesali, Maryam Zareei, Zahra Hajinaghibali Hessari

**Affiliations:** 1 Department of Endocrinology and Female Infertility, Reproductive Biomedicine Research Center, Royan Institute for Reproductive Biomedicine, ACECR, P.O. Box: 16656-59911, Number 12, East Hafez Avenue, Bani Hashem Street, Resalat Highway, Tehran, Iran; 2 Department of Basic and Population Based Studies in NCD, Reproductive Epidemiology Research Center, Royan Institute, ACECR, Tehran, Iran; 3 Department of Embryology, Reproductive Biomedicine Research Center, Royan Institute for Reproductive Biomedicine, ACECR, Tehran, Iran

**Keywords:** Polycystic ovary syndrome, L-carnitine, IVF/ICSI cycles, Oocyte quality, Weight loss, Lipid profile

## Abstract

**Objective:**

To investigate the effect of L-carnitine supplementation during the controlled ovarian stimulation (COS) cycle with antagonist protocol in patients with polycystic ovary syndrome (PCOS) diagnosis undergoing IVF/ICSI treatment.

**Methods and materials:**

This was a double-blind clinical trial study including 110 patients with PCOS attended to Royan Institute between March 2020 and February 2023. At the beginning of the COS cycle, the eligible patients were allocated into two groups randomly according to the coding list of the drugs prepared by the statistical consultant. In the experimental group, patients received 3 tablets daily (L-carnitine 1000 mg) from the second day of menstruation of the previous cycle until the puncture day in the cases of freeze-all embryos (6 weeks) or until the day of the pregnancy test (8 weeks) in fresh embryo transfer cycle. In the control group, patients received 3 placebo tablets for the same period of time. Weight assessment and fasting blood sugar and insulin tests, as well as serum lipid profile were also measured at the baseline and ovum pick-up day. The results of the COS cycle as well as the implantation and pregnancy rates were compared between groups.

**Results:**

Finally, 45 cases in L-carnitine group versus 47 cases in the placebo group were completed study per protocol. Data analysis showed that the two groups were homogeneous in terms of demographic characteristics and baseline laboratory tests and severity of PCOS. There is no statistically significant difference in terms of the oocyte recovery ratio and oocyte maturity rate, and the number and quality of embryos, as well as the rates of the fertilization, chemical and clinical pregnancy between groups. However, the means of weight (*P* < 0.001) and serum levels of fasting blood sugar (*P* = 0.021), fasting insulin (*P* = 0.004), triglyceride (*P* < 0.001) and cholesterol (*P* < 0.001), LDL (*P* < 0.001) have significantly decreased in women after consuming L-carnitine supplementation.

**Conclusion:**

The oral intake of L-carnitine during COS in PCOS women for 6 weeks had no effect on COS and pregnancy outcomes. However, taking this supplement for 6 weeks has been associated with weight loss and improved lipid profile and serum glucose.

**Trial registration:**

The study was registered in the Clinicaltrials.gov site on December 17, 2020 (NCT04672720).

## Background

One of the most common endocrine disorders in reproductive-aged females is polycystic ovary syndrome (PCOS), which due to the multifactorial nature for its pathophysiology, including genetic, life-style, and environmental factors, clinical specialists face multitudinous challenges to treat these patients with infertility problem. There are a wide variety of medicines that reduce insulin resistance, antioxidants, and anti-inflammation drugs have been studied and recommended for the treatment of these patients prior to starting infertility treatment cycles [1].

Carnitine is available in the form of two stereoisomers, L-carnitine, which is the biologically active form, and D-carnitine, the biologically inactive form [2]. L-carnitine is widely accessible as a nutritional supplement which plays a vital role in the stability of mitochondrial membranes and increasing energy storage in organelles and preventing apoptotic cell death [3]. Also, the effect of L-carnitine on weight loss, glucose metabolism and insulin function, oxidative stress and fatty acid metabolism has also been reported [4]. Its possible mechanism of action includes increasing the amount of excess acyl groups leaving the mitochondria of insulin-responsive tissues and facilitating the transfer of free fatty acids into the mitochondrial matrix [5, 6].

The use of L-carnitine in the treatment of insulin resistance has been suggested due to the role of the accumulation of acyl-CoA derivatives in causing insulin resistance [7]. In human studies and animal models, L-carnitine supplementation shows that it improves glucose homeostasis parameters, especially in insulin-resistant states [7]. In a number of previous studies, it has been reported that the circulating amount of total and free L-carnitine in women with PCOS has significantly decreased and this decrease has been associated with markers of hyperandrogenemia and hyperinsulinemia [6]. In addition to these recent researches, the insufficient amount of serum L-carnitine has been proposed as a factor causing insulin resistance in chronic metabolic stress situations such as type II diabetes and obesity [7].

On the other hand, frequent ovarian stimulation in PCOS patients reduces the amount of mitochondrial DNA and is associated with an increase in the amount of 8-hydroxydeoxyguanosine in the oocytes [7, 8]. Moreover, a decrease in the expression of the mitochondrial transcription factor A gene and the occurrence of more oocytes with distributed, decentralized mitochondria have also been reported in these patients [7]. As a result, it seems that in PCOS patients with a history of frequent inappropriate response to ovarian stimulation, the use of L-carnitine supplements can be effective in increasing the oocyte quality and reproductive outcomes [9].

Furthermore, daily consumption of up to 4000 mg of L-carnitine has been found in past studies to have no side effects. Except for dialysis patients, consumption of 50 to 350 mg per kilogram of body weight has been safe in human studies [10]. In the field of infertility treatment for the first time, Ismail et al. (2014), reported that the simultaneous use of L-carnitine and clomiphene citrate significantly improved both ovulation quality and cumulative pregnancy rate in patients with PCOS resistant to clomiphene [9]. The idea of adding L-carnitine in the follicular phase was presented with the assumption that L-carnitine inhibits reactive oxygen species and acts as a destroyer of harmful oxidative stress substances accumulated by previous cycles of ovulation induction [11]. Thereupon, some prospective studies or clinical trials have been conducted in this field, with conflicting results. The present study was one of the first studies registered on the clinicaltrials.gov site, which was designed with the aim of investigating the effect of using L-carnitine supplements during the ovarian stimulation cycle with antagonist protocol in patients with PCOS on the IVF/ICSI outcome.

## Methods

This randomized double-blind clinical trial was implemented at female infertility and reproductive endocrinology department of the Royan institute from March 2020 to February 2023. The patients with PCOS-related infertility aged between 20 and 37 who had indications for IVF/ICSI treatment and written consent to participate in the study were enrolled in the study. The PCOS diagnosis was based on the Rotterdam’s criteria and following a complete history taking, physical examination and a paper documented complete infertility work-up [12]. The following items were considered as exclusion criteria: 1-diagnosis of hyperprolactinemia, diabetes mellitus, epilepsy or any chronic heart, lung, liver or kidney disease, 2- treatment with special diet, medication supplement (ovuboost), metformin before or during ovarian stimulation, 3- history of pelvic surgery on ovaries and uterus, 4- presence of submucosal and intramural fibroids larger than 5 cm or the presence of uterine polyps and congenital uterine malformations, 5- the severe male factor infertility.

Prior to the onset of ovarian stimulation, the eligible patients were allocated randomly into two groups according to the blocked randomization method. The methodologiest provided the clinical specialist with an English three-letter code according to the randomization list, which corresponds to a code on the medicine’s box, then the specific box was delivered to the patient. The appearance of the placebo tablets was indistinguishable in color, shape, size, and smell from L-carnitine tablets, besides whole medicine boxes were identical, therefore the clinical specialist who was involved in treatment and follow-up as well as the patients did not know the type of drug and the study was conducted in a double-blind manner.

The COS procedure in all the study participates was same by using a flexible regimen of GnRH antagonist. In the experimental group, the women received three tablets of L-carnitine daily (L-carnitine® tablet 1000 mg, Karen Pharmaceutical Company, Iran) from day 2 of the previous menstrual cycle until the puncture day in the cases of freeze-all embryos (6 weeks) or until the day of the pregnancy test (8 weeks) in fresh embryo transfer cycle. The patients in the control group will receive three placebo tablets for the same period of time. All the placebo tablets were produced by the Karen Pharma Company (Tehran, Iran), which is approved by the Food and Drug Administration of Iran. Ovarian stimulation was initiated with a dose of 150 units of rFSH (Gonal -F: Serono Laboratories Ltd., Geneva, Switzerland), from the second or third day of the spontaneous or discontinued progesterone menstrual cycle and the monitoring transvaginal ultrasound was performed and then if the follicle ≥13 mm was observed, the injection of GnRH antagonist (Cetrotide®, 0.25 mg cetrorelix acetate*,* Serono, Inc) was started and continued until the oocyte triggering day. From the seventh day of the cycle, the dose of rFSH was adjusted according to the rate of ovarian response by vaginal ultrasonography two days in advance. If at least two follicles 18 mm in size or more were observed, the final oocyte maturation was done. In cases where, based on the evidence of ultrasound and the serum estradiol level on the day of the oocyte trigger, there is a risk of ovarian hyperstimulation syndrome (OHSS), the final trigger was performed with the administration of triptorelin 0.2 mg (Decapeptyl®) and otherwise with 250 micrograms of Ovidrel®. Approximately 34–36 hour after that the oocyte retrieval was carried out and subsequently ICSI or ICSI/IVF was done with standard procedure. The quality of embryos were determined according to the number of cells, the amount of fragmentation, the variation in cell size and overall symmetry (perfect, moderately asymmetric, and severely asymmetric in size and shape of the cells at Day 2 or Day 3 after oocyte retrieval: Excellent (Day 3: 6–8 even size blastomeres with ≤10% fragmentation), Good (Day 3: 6–8 even or uneven size blastomeres with 10–20% fragmentation), Poor (Uneven and few blastomeres with > 20% fragmentation). The type of embryo transfer was determined by the clinician based on the risk of OHSS, therefore in the presence of the OHSS risk, the freeze-all policy has been applied. In cases of fresh embryo transfer, progesterone suppository (Cyclogest®; Actavis, Barnstaple, UK) twice a day was prescribed for luteal phase support. In the frozen embryo transfer (FET) cycles, the endometrium was prepared by hormonal protocol using a GnRH pretreatment as described previously.

### *Clinical laboratory* tests

Hormonal and biochemical assessments were carried out for all patients participating in the study in the Royan Institute laboratory. The basal serum levels of FSH and luteinizing hormone (LH), anti-Mullerian hormone (AMH), thyroid stimulating hormone (TSH) as well as free testosterone and dehydroepiandrosterone sulphate (DHEAS) concentrations were measured in a venous blood sample collected from all participants on the day 2 or 3 of their menstrual cycle. AMH level, FSH and LH concentrations were measured by Electrochemiluminescence immunoassay (ECLIA) using fully automated COBAS E-601 analyzer and a commercial kit (Roche Diagnostics GmbH, Mannheim, Germany). The assessment of free testosterone and DHEAS concentrations was performed by a commercial enzyme-linked immunosorbent assay) ELISA) kit (Abbott Healthcare Pvt. Ltd., Ireland) with inter- and intra-assay coefficient of variations (CVs) less than 5%. Serum estradiol level on the oocyte triggering day was measured using an ECLIA method and a commercial kit (Roche).

In addition, the markers of insulin metabolism (fasting glucose and insulin) and lipid profiles (low-density lipoprotein (LDL), high-density lipoprotein (HDL), cholesterol and triglyceride) were measured at the baseline as well as 6 weeks later. The blood Samples were then kept at − 80 °C until the day of analysis. Biochemistry tests are analyzed using fully automated COBAS C-501 device by a commercial kit (Roche). All inter- and intra-assay CVs for measurements were less than 5%.

### The study outcomes

The primary outcome was considered oocyte maturity rate that was defined as the ratio of metaphase II (MII) oocytes to the number of retrieved oocytes. The secondary outcomes includes.

The fertilization rate (the number of 2PNs divided by the total number of MII oocytes), quality of obtained embryos, implantation rate (the number of intrauterine gestational sacs divided by transferred embryos), as well as clinical pregnancy rate (by detecting fetal heartbeats two weeks following the positive β hCG) and ongoing pregnancy rate (a pregnancy documented by ultrasound over 20 gestational weeks that showed the presence of normal fetus). Meanwhile, weight changes as well as some biochemical tests before and after supplement administration were investigated.

### Statistical analysis

It was estimated the sample size of 55 cases in each arm to be appropriate to the detection of a 10% increase in oocyte maturity rate as our primary outcome after L-carnitine administration with 80% power at 5% alpha level. Statistical analysis was performed using SPSS software (version 22; Inc., Chicago, IL, USA). All data were shown as mean ± Standard deviation (SD) and number (percent). Data were analyzed using the 2-tailed student t-test and Chi-square test between groups, where appropriate. The changes of the investigated variables before and after taking the supplement in each group were calculated using paired t-test. A *p*-value < 0.05 was considered statistically significant.

## Results

**Fig. 1:**
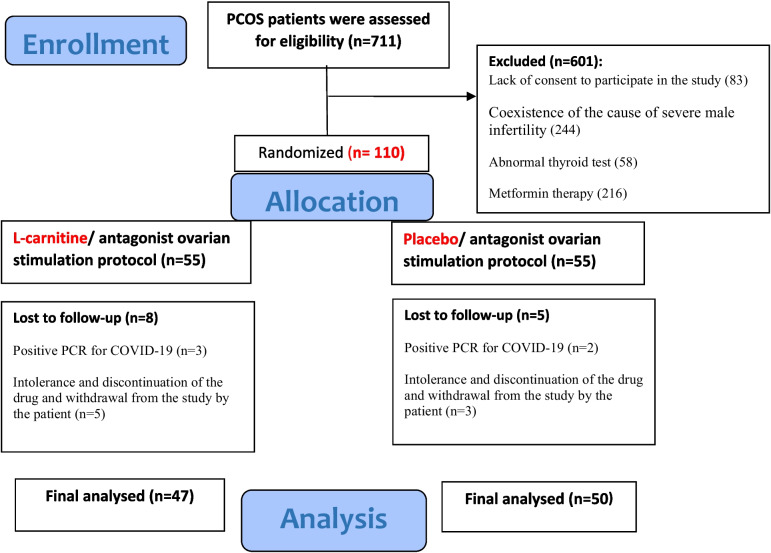
Study flowchart

During the research period, 711 women were evaluated for enrollment and finally, 110 eligible patients were included in the study. In the follow-up, only 97 patients completed the study according to the treatment cycle protocol, and statistical analysis was performed between L-carnitine (*n* = 47) and control (*n* = 50) groups (Fig. 1).

Baseline characteristics including female age (*P* = 0.55), type (*P* = 0.36), duration (*P* = 0.77) and cause of infertility (*P* = 0.10), body mass index (BMI) (*P* = 0.96) as well as basal serum levels of FSH (P = 0.96) and LH (*P* = 0.16), AMH (*P* = 0.26) and TSH (*P* = 0.20) were similar between the two groups. In addition, there was no significant difference between the two groups in terms of the baseline levels of lipid profile markers including cholesterol (*P* = 0.89), triglyceride (*P* = 0.06), LDL (*P* = 0.71), HDL (*P* = 0.26), as well as FBS (*P* = 0.43), fasting insulin (*P* = 0.24), free testosterone (*P* = 0.43) and DHEA concentrations (*P* = 0.35). The different phenotypes of PCOS have been compared between groups, and the studied groups were almost similar in this regard (*P* = 0.16). Moreover, the mean number of previous intrauterine insemination (IUI) cycles was similar between the two groups (Table 1).

**Table 1:** Baseline characteristics of patients in the ‘L-carnitine’ group versus control group

Variable		L-Carnitine group *n* = 47	Placebo group *n* = 50	*p*-value*
Age (year)		29.8 (4.0)	30.4 (4.3)	0.55
Body mass index (Kg/m^2^)		27.4 (5.2)	27.4 (3.7)	0.96
FSH (IU/L)		5.7 (1.9)	5.7 (1.7)	0.96
LH (IU/L)		6.2 (3.9)	7.2 (3.1)	0.16
TSH (mIU/L)		1.5 (0.9)	1.8 (0.9)	0.20
AMH (ng/mL)		7.2 (3.7)	8.1 (4.4)	0.26
DHEA (μg/dL)		202.8 (119.4)	176.2 (132.2)	0.35
Free testosterone (nmol /L)		2.50 (1.6)	1.8 (1.1)	0.13
FBS (mg/dL)		94.1 (12.0)	92.4 (8.9)	0.43
Insulin (uIU/mL)		11.5 (5.3)	10.1 (5.6)	0.24
Cholesterol (mg/dL)		161.1 (38.7)	162.1 (35.4)	0.89
Triglyceride (mg/dL)		141.4 (50.8)	120.7 (52.1)	0.06
LDL (mg/dL)		98.6 (32.6)	96.3 (27.1)	0.71
HDL (mg/dL)		43.5 (14.0)	46.7 (12.4)	0.26
Infertility duration (year)		6.8 (3.9)	6.5 (4.0)	0.77
Type of infertility	Primary	40 (85.1)	39 (78.0)	0.36
	Secondary	7 (14.9)	11 (22.0)	
Cause of infertility	PCOS	26 (55.3)	36 (72.0)	0.10
	PCOS& male factor	21 (44.7)	14 (28.0)	
Phenotype^α^	A	23 (43.9)	18 (36.0)	0.16
	B	6 (12.8)	3 (6.0)	
	C	12 (25.5)	20 (40.0)	
	D	6 (13.8)	9 (18.0)	
No. of previous IUI		2.3 (1.4)	2.0 (0.95)	0.46

Values are presented as the mean ± Standard deviation (SD) and number (percent). * Obtained by independent t test and chi square test. Statistically significant level was 0.05

^α^ Phenotype A (full-fledged polycystic ovary syndrome (PCOS): HA + OD + PCO) includes hyperandrogenism (HA) (clinical or biochemical), ovulatory dysfunction (OD), and polycystic ovaries (PCO) (HA + OD + PCO). Phenotype B (non-PCO PCOS: HA + OD). Phenotype C (ovulatory PCOS: HA + PCO). Phenotype D (non-hyperandrogenic PCOS: OD + PCO) [13]

According to Table 2, the comparison of ovarian stimulation cycle features in the two groups showed that there was no statistically significant difference between groups in terms of the total number of used gonadotropins (*P* = 0.72) and GnRH antagonist (*P* = 0.30) ampules, duration of stimulation (*P* = 0.48) and serum estradiol level on the day of oocyte triggering (*P* = 0.75). Furthermore, no statistically significant differences were observed regarding ovarian response markers including total number of observed follicles on the oocyte trigger day, total number of retrieved oocytes and the number of MII oocytes as well as oocyte recovery ratio, oocyte maturity and fertilization rates between the two groups (*P* > 0.05). As shown in Table 2, characteristics of obtained embryos in patients were compared between groups. There was no significant difference between groups regarding the number and quality of obtained embryos (*p* > 0.05).

**Table 2:** The comparison of ovarian stimulation cycle outcomes between two groups

Variable	L-carnitine group *n* = 47	Placebo group *n* = 50	*p*-value*
Estradiol level on oocyte trigger day (pg/ml)	2625.7 (1363.1)	2536.4 (1410.1)	0.75
Total no. of used gonadotropin ampules	21.8 (7.6)	21.2 (8.1)	0.72
Stimulation duration (day)	10.2 (2.1)	9.9 (1.9)	0.48
Antagonist duration (day)	4.4 (1.2)	4.1 (1.0)	0.30
Total no. of observed follicles on oocyte trigger day	16.5 (6.2)	16.5 (5.0)	0.96
No. of follicles > 18 mm on oocyte trigger day	2.7 (1.7)	2.1 (1.5)	0.09
No. of retrieved oocytes	12.4 (6.2)	11.6 (4.7)	0.49
No. of MII oocytes	10.5 (4.6)	9.9 (4.7)	0.53
Oocyte maturity rate	0.86 (0.13)	0.83 (0.19)	0.44
Oocyte recovery ratio	0.63 (0.18)	0.59 (0.20)	0.23
Fertilization rate	0.70 (0.23)	0.73 (0.23)	0.37
Endometrium thickness on oocyte trigger day (cm)	10.0 (1.9)	9.5 (1.5)	0.10
All-freeze cases	40 (85.1)	41 (82)	0.68
Total no. of obtained embryos	9.9 (6.2)	10.2 (6.8)	0.77
No. of top-quality embryos	6.1 (6.0)	5.0 (4.7)	0.31
No. of frozen embryos (cleavage stage)	8.0 (6.1)	7.9 (5.8)	0.89

Values are presented as the mean ± Standard deviation (SD) and number (percent). * Obtained by independent t test and chi square test. Statistically significant level was 0.05. *MII* metaphase II; *No.* number

In the follow-up of ET cycles for patients, it was found that 5 patients (10.6%) in the l-carnitine group and 3 patients (6%) in the control group have not referred for FET cycles until the time of data analysis (*P* = 0.65). The number and percentage of cases of fresh and frozen ET cycles in two groups were compared, and there was no statistically significant difference between the two groups in this respect. Comparison of both fresh and frozen ET cycles’ pregnancy outcomes between groups revealed that there were no significant differences in the implantation, clinical and ongoing pregnancy rates according to the type of ET cycles (Table 3).

**Table 3:** The comparison of embryo transfer cycles outcome between two groups

Variable	L-carnitine group *n* = 47	Placebo group *n* = 50	*p*-value*
The number of cases that have not referred for frozen embryo transfer until the time of data analysis	5 (10.6%)	3 (6%)	0.65
No. of transferred embryos in fresh cycles	2.1 (0.37)	2.2 (0.44)	0.71
No. of transferred embryos in FET cycles	2.0 (0.27)	2.0 (0.37)	0.38
Implantation rate in fresh ET cycle (mean ± SE)	0.25 (0.25)	0.26 (0.11)	0.95
Clinical pregnancy rate/ fresh ET	1/7 (14.3%)	3/9 (33.3%)	0.58
Ongoing pregnancy rate/fresh ET	1/7 (14.3%)	2/9 (22.2%)	0.68
Implantation rate in FET cycle	0.54 (0.35)	0.52 (0.27)	0.89
The clinical pregnancy rate /FET cycle	17 /35 (48.5%)	20 /38 (52.6%)	0.71
The ongoing pregnancy rate/ FET cycle	15 / 35 (42.8%)	19 / 38 (50.0%)	0.70
Cumulative ongoing pregnancy rate/total ET cycles	16 /42 (43.2%)	21/ 47 (56.8%)	0.67

Values are presented as the mean ± Standard deviation (SD) and number (percent).*Obtained by independent t test and chi square test. Statistically significant level was 0.05. *No.* number; *FET* frozen embryo transfer

Six weeks after taking supplements in the L-carnitine group, a statistically significant decrease was observed in terms of weight (*P* < 0.001), lipid profile markers including cholesterol (*P* < 0.001), triglyceride (*P* < 0.001), LDL (*P* < 0.001), as well as FBS (*P* = 0.021) and fasting insulin (*P* = 0.004) compared to baseline levels. On the other hand, in the placebo group, no significant changes in terms of weight, serum levels of FBS, insulin concentrations and lipid profile were found (Table 4).

**Table 4:** The changes in weight and clinical laboratory tests after 6 weeks in the studied groups

Variable		Baseline	Puncture day	Changes	*p*-value*
**L-carnitine group**	Weight (kg)	70.0 (12.0)	67.3 (9.6)	−2.65	< 0.001
	FBS (mg/dL)	94.1 (12.0)	90.2 (6.7)	−3.95	0.021
	Fasting Insulin (uIU/mL)	11.5 (5.3)	10.0 (4.8)	−1.46	0.004
	Cholesterol (mg/dL)	161.1 (38.7)	142.7 (32.9)	−18.3	< 0.001
	Triglyceride (mg/dL)	141.4 (50.8)	117.70 (43.9)	−24.4	< 0.001
	LDL (mg/dL)	98.6 (32.6)	82.0 (28.1)	−16.6	< 0.001
	HDL (mg/dL)	43.5 (14.0)	46.8 (13.5)	3.3	< 0.001
**Control group**	Weight (kg)	70.6(10.2)	70.3 (10.5)	−0.35	0.56
	FBS (mg/dL)	92.4 (8.9)	92.3 (9.0)	0.10	0.89
	Fasting insulin (uIU/mL)	10.1 (5.6)	10.4 (4.7)	0.39	0.26
	Cholesterol (mg/dL)	162.1 (35.4)	161.9 (31.7)	−0.18	0.94
	Triglyceride (mg/dL)	120.7 (52.1)	125.1 (46.9)	−4.43	0.25
	LDL (mg/dL)	96.3 (27.1)	95.4 (28.9)	−0.83	0.73
	HDL (mg/dL)	46.7 (12.4)	47.2 (11.5)	0.46	0.52

Values are presented as the Mean ± Standard deviation (SD). * Obtained by paired t test. Statistically significant level was 0.05

## Discussion

The results of the present study showed that adding L-carnitine to the ovarian stimulation protocol did not have a beneficial effect on the number of retrieved and mature oocytes, the fertilization rate, and the number and quality of embryos compared to the placebo group. Also, the rates of implantation, as well as chemical and clinical pregnancy, were similar between the study groups. Although the means of weight and serum levels of fasting blood sugar and insulin, triglyceride and cholesterol, LDL were significantly reduced after 6 weeks of consumption in women consuming L-carnitine.

There are several studies which have reported the valuable effects of L-carnitine in PCOS women in terms of weight loss, balancing the fat profile, as well as regulating menstruation and reducing hirsutism [14, 15]. Another possibility is that the antioxidant activity of L-carnitine can improve and maintain mitochondrial function in oocytes and lead to recovery of good quality oocytes with high growth capacity [16]. Based on the aforementioned theories, we hypothesized that adding L-carnitine to the stimulation protocol may improve ART outcomes in infertile women with PCOS; however, the study outcomes did not support this hypothesis.

In line with the results of our study, Sheida et al., investigated the effect of adding L-carnitine to the antagonist ovarian stimulation protocol in IVF/ICSI cycles and their results demonstrated that the administration of L-carnitine during ovulation induction in PCOS women could not improve the quality of the retrieved oocytes and pregnancy rate [17]. Contrary to our findings, the result of an RCT that evaluated the effect of adding L-carnitine to clomiphene citrate on pregnancy outcome in ovarian stimulation and IUI cycles showed improvement in ovulation and cumulative pregnancy rates in clomiphene-resistant women with PCOS. Also, the number of follicles larger than 17 mm, the concentration of estradiol and the thickness of the endometrium were significantly higher in the L-carnitine group [9]. In a similar randomized clinical trial with a smaller sample size, Abdelfattah et al., confirmed that adding L-carnitine to clomiphene citrate in the follicular phase and continuing administration into the luteal phase in clomiphene-resistant patients can improve ovulation quality and the rates of chemical and clinical pregnancy [18]. In contrast, another RCT showed that the addition of L-carnitine to clomiphene citrate in women with PCOS could improve the number and quality of mature oocytes; however, the pregnancy rate did not change significantly [19].

In view of the fact that obesity and insulin resistance are so common in PCOS women, Sharkey and colleagues through an RCT evaluated the effects of L-carnitine supplementation on reproductive outcomes in obese PCOS women. It was concluded that L-carnitine supplementation are associated with significant improvements in menstrual regularity, ovulation and pregnancy rates in compared to the metformin and placebo groups in these women [20]. Likewise, another RCT confirmed the beneficial effect of L-carnitine combined with letrozole on ovulation, chemical and clinical pregnancy rates in these women [17]. In a before-and-after study with large sample size (214 infertile PCOS women with a history of previous IVF failure) has been reported that the embryo quality on days 3 and 5 were significantly increased after L-carnitine administration; however, no statistically noticeable differences in the total number of retrieved and MII oocytes and fertilization rate before and after using L-carnitine supplementation were found [21]. The discrepancies can be due to different design of the studies and also the duration of L-carnitine consumption, in such a way that it was on average 82 days in the Kitano and co-workers’ study, while in the present study it was consumed for 6 weeks (42 days) by almost 85% of patients.

In our study, consumption of L-carnitine for 6 weeks was associated with an average weight loss of 2.5 kg, as well as a significant improvement in the profiles of glucose and lipid metabolism. In the previous studies, it has been reported that L-carnitine supplementation improves PCOS symptoms through diminishing the insulin resistance and subsequently reducing blood glucose levels [14]. It has also been shown that adding L-carnitine to the diet of PCOS women may regulate the ratio of male and female hormones and reduce the effects of hyperandrogenism [6]. In addition, there is evidence about improving ovulation and pregnancy rate with the use of L-carnitine in PCOS women [9]. However, in the present study, we did not observe improvement in the results of ovarian stimulation and embryo quality.

According to the NCEP ATP III definition, metabolic syndrome is present if three or more of the following five criteria are met: waist circumference greater than 40 in. (in men) or 35 in. (in women), blood pressure greater than 130/85 mmHg, fasting triglyceride level (higher than 150 mg/dL), HDL level less than 50 mg/dL, and fasting blood sugar more than 100 mg/dL [22]. Based on the recorded data, none of the patients included in the present study had 3 of the 5 diagnostic criteria of metabolic syndrome. It is possible that a longer period of time or a specific population of PCOS patients with excessive obesity or metabolic syndrome is needed to observe the positive effects of L-carnitine supplementation on the oocyte quality, so further clinical trials considering these issues are suggested. It should be designed at least three months before the start of the ART cycle in the population of PCOS patients with the problem of excessive obesity and/or with a history of inappropriate ovarian response.

To the best of our knowledge, this is the first double-blind randomized clinical trial comparing the effect of L-carnitine on the results of IVF/ICSI cycles in PCOS women with placebo. Due to financial and time constraints, it was not possible to take L-carnitine supplementation for at least 3 months before the ART cycle, so it can be the limitation of the present study.

## Conclusions

By virtue to the present findings, the oral intake of L-carnitine during ovulation induction in PCOS women for 6 weeks had no effect on the number and quality of the obtained oocytes and embryos and the clinical pregnancy rate following FET cycles. Certainly, taking the supplements for 6 weeks has been associated with weight loss and improved lipid profile and serum glucose. It is suggested to use a larger sample size to evaluate the effect of different doses and a longer period (12 weeks) of L-carnitine supplementation on the outcomes of ART cycles in PCOS women with excessive obesity or with a history of frequent inappropriate ovarian responses.

## Data Availability

The datasets used and/or analyzed during the current study are available from the corresponding authors on reasonable request.

## Abbreviations

AMH: Anti-müllerian hormone
ART: Assisted reproductive technology
COS: Controlled ovarian stimulation
DHEAS: Dehydroepiandrosterone sulphate
ECLIA: Electrochemiluminescence immunoassay
ELISA: Enzyme-linked immunosorbent assay
FET: Frozen embryo transfer
FBS: Fasting blood sugar
GnRH: Gonadotropin-releasing hormone
HCG: Human chorionic gonadotropin
HDL: High-density lipoprotein
IVF/ICSI: in vitro fertilization/intracytoplasmic sperm injection
LDL: Low-density lipoprotein
LH: Luteinizing hormone
OHSS: Ovarian hyperstimulation syndrome
PCOS: Polycystic ovarian syndrome
rFSH: Recombinant follicle stimulating hormone
SD: Standard deviation
